# Study on Strength, Water Stability, Shrinkage, and Microstructure of CFB Slag Modified Cement Stabilized Clay

**DOI:** 10.3390/ma14237460

**Published:** 2021-12-05

**Authors:** Mingkai Zhou, Xinyue Liu, Xiao Chen, Peng Gao

**Affiliations:** 1 State Key Laboratory Silicate Materials for Architecture, Wuhan University of Technology, Wuhan 430070, China; silicate@whut.edu.cn; 2 School of Materials Science and Engineering, Wuhan University of Technology, Wuhan 430070, China; X13667106507@163.com (X.L.); 13572186187@163.com (P.G.)

**Keywords:** cement, base, CFB slag, shrinkage

## Abstract

Circulating fluidized bed slag (CFBS) is an industrial waste produced by coal combustion in power plants. To explore the application of CFB slag in cement-stabilized bases, this paper studies the influence of different dosage of CFBS on the mechanics, water stability, and shrinkage of cement-stabilized soil using laboratory experiments. The hydration activity and interface morphology of CFBS in cement-stabilized clay were observed using XRD and SEM. The improvement mechanism of CFBS on the performance of cement-stabilized clay was revealed. The results indicated that, compared with cement-stabilized clay, cement–CFBS-stabilized clay exhibited better mechanical and water stability, and significantly inhibited the shrinkage deformation of cement-stabilized clay. When the addition of CFBS was 70%, cement–CFBS-stabilized clay had the best mechanics and durability. Microscopic tests show that CFBS contains more active silicon aluminum oxide, which is easily dissolved and the hydration of which produces more gel products, so the mixture structure is denser, the strength is improved, and water does not easily evaporate; it has the characteristics of micro expansion which compensates for dry shrinkage deformation.

## 1. Introduction

Since the 20th century, cement-stabilized clay has been widely used in pavement bases and sub-bases, as cement greatly improves the mechanical properties of semirigid bases, as well as the deformation resistance, permeability, and durability of pavement structures [1,2,3]. However, due to the environmental impact and changes in the moisture content of mixtures, the shrinkage deformation of semirigid bases can occur, and shrinkage cracking is the main problem that limits its wider application [4,5,6]. Moreover, there is lower cement dosage and the strength is low in semirigid base materials. Therefore, it is of great significance to conduct in-depth research on improving the strength and water stability, shrinkage of cement-stabilized clay [7,8].

At present, there are many studies on industrial waste and cement to stabilize base materials. These studies on the properties of cement-stabilized base materials mainly include the following three improvement mechanisms: (1) The micro-aggregate effect: Small particle-size aggregates filled soil particle pores; for example, the stiffness of cement-stabilized clay can be improved by using the particle characteristics of sand [9]. Harder sand particles that have been bonded to a cemented clay matrix can lead to an increased hardness. (2) The volcanic ash reaction: Reaction of active components with calcium hydroxide to produce hydrated calcium silicate, hydrated calcium aluminate, improving the connection among particles. Industrial waste, such as magnesium slag, sintered limestone, domestic waste incinerator slag, and CFB-fly-ash [10,11], mainly rely on the hydration reaction of their active components to produce more cement products, thereby improving the characteristics of cement-stabilized base materials. (3) The micro-crystal nucleus effect: Provide crystal nucleus of hydration products to accelerate cement hydration. For example, the addition of milled slag [12,13] to cement-stabilized soil can provide crystal nuclei and accelerate cement hydration however, there are some drawbacks. First, due to the commonality of common industrial wastes, such as sand, sintered limestone, and slag, the recovery rate is high and continues to grow. Second, the activity of ordinary industrial waste is relatively low although it can improve the characteristics of cement-stabilized soil to some degree, the effect is not ideal. Therefore, this paper uses circulating fluidized bed slag (CFBS) to improve the characteristics of cement-stabilized clay.

CFBS is the waste slag discharged from the bottom of a circulating fluidized bed boiler. It has high activity and is self-hardening, but it is hydrophilic [14,15,16] and contains free calcium oxide and anhydrite. The use of CFBS in cement and concrete may lead to poor durability and an unstable volume, which is the main reason for restricting the resource utilization of CFBS [14]. In China, 90 million tons of CFBS are discharged annually, but resource utilization rate is low. Previous studies have shown that CFBS can be used as a pavement base, mainly for soil treatment. As the particle characteristics of CFBS are similar to those of sand, it has a continuous gradation and fills the clay; it contains also more burnt clay minerals and a certain amount of f-CaO, II-CaSO_4_ [17]. After mixing with water, a volcanic ash reaction occurs between its components and there is a certain degree of micro expansion. Therefore, using CFBS to directly improve the adverse characteristics of cement-stabilized clay may have promising prospects.

This study carried out a series of mechanical, water stability, and shrinkage tests of cement–CFBS-stabilized clay. X-ray diffraction (XRD) and scanning electron microscopy (SEM) were used to reveal the improvement principle of CFBS on cement-stabilized clay, from macroscopic structure to microscopic product formation.

## 2. Materials and Methods

### 2.1. Materials

**Figure 1:**
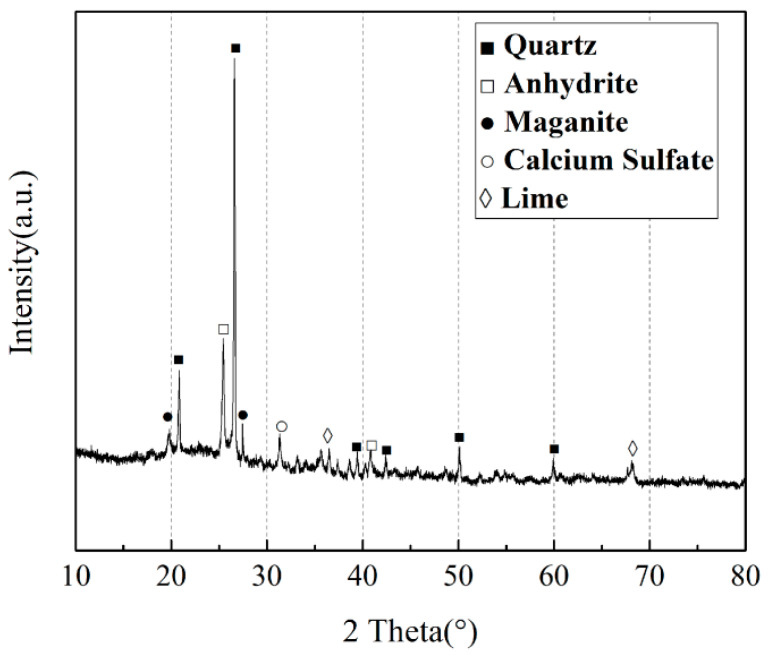
The XRD patterns of CFBS.

**Figure 2:**
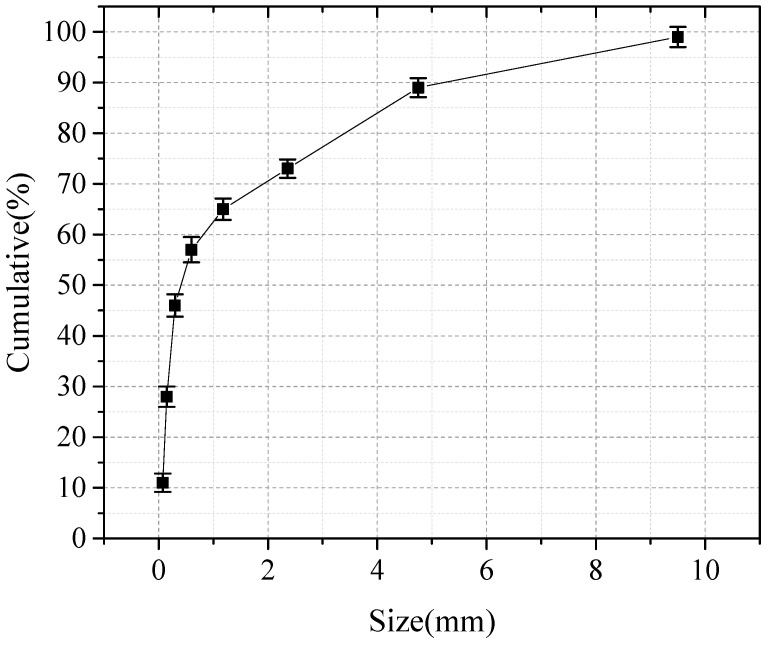
Particle characteristics of CFBS.

The CFBS was supplied from Jinneng Datuhe Gangue Power Plant (Shanxi, China) Cement was supplied from Zhuoyue Cement Co., Ltd. used in the tests were taken from Shanxi Province, China. The chemical and mineral compositions of CFBS are shown in Figure 1 and Table 1, and the particle size distribution is shown in Figure 2. The performance of cement is shown in Table 2. The liquid limit of the soil was 27%, and the plasticity index was 8, making it a low-liquid limit clay.

### 2.2. Methods

**Figure 3:**
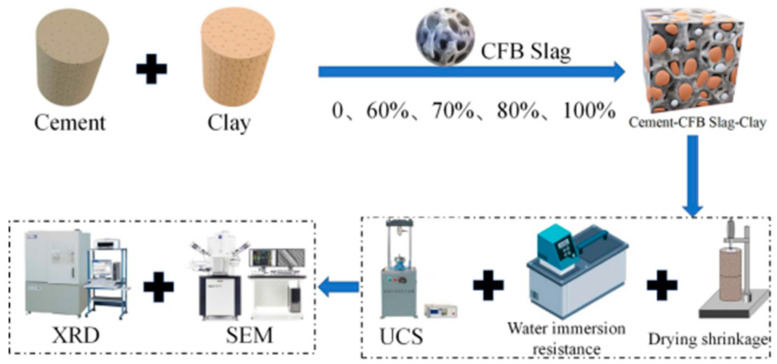
Experimental process diagram.

To study the effect of different content of CFBS on the performance of cement stabilized clay, the following mix ratio was set up. The cement content was 6% and clay with the same quality of CFBS instead. The addition of CFBS was 0, 60%, 70%, 80%, and 100%, numbered as CS0, CS6, CS7, CS8, and CS10. According to the compaction test of T0804-1994 in Chinese standard (JTG E51-2009), the maximum dry density (MDD) and the optimum moisture content (OMC) of the mixture were determined. The details are shown in Table 3. The experimental process is shown in Figure 3

#### 2.2.1. Strength Tests

The mixture was pressed into a Φ100 mm × 100 mm cylinder with 98% compaction. The pressed samples were solidified under standard curing conditions (RH = 98%, T = 20 °C). The specimen was immersed in water 24 h before the strength test was carried out until the test age. The unconfined compressive strength (UCS) and splitting strength (SS) of specimens were tested according to the experimental methods of T0805-1994 and T0806-1994 in Chinese standard (JTG E51-2009). The test period was 7 days, 28 days, 90 days, and 180 days (7d, 28d, 90d, and 180d).

#### 2.2.2. Water Stability Tests

The mixture was pressed into a Φ100 mm × 100mm cylinder with 98% compaction. The pressed samples were solidified under standard curing conditions (RH = 98%, T = 20 °C). After that, soaking curing was carried out, and the ages were 0 days, 1 days, 2 days, and 4 days (0d, 1d, 2d, and 4d) respectively. The UCS of the specimens was measured at 7d. In particular, the UCS and UCS retention coefficients were determined by comparing the UCS of the 1d, 2d, and 4d immersion specimens with the UCS of the 0d immersion specimens.

#### 2.2.3. Shrinkage Tests

**Figure 4:**
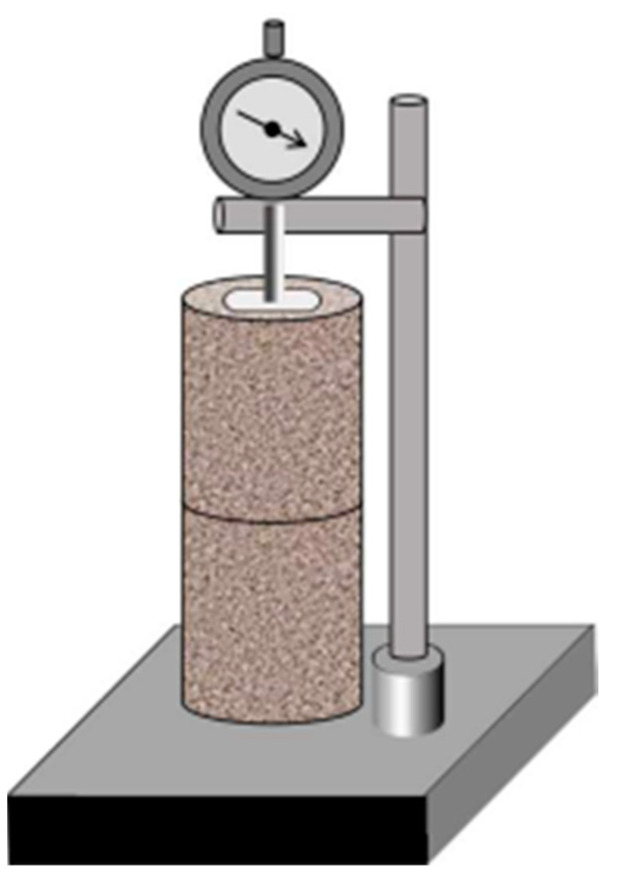
Contraction instrument model.

The size of the pressed sample is Φ100 mm × Φ100 mm, and the compactness is 98%. The formed specimens were sealed in plastic bags and placed in a health room at a constant temperature (20°C ± 2 °C) with a constant humidity (98% relative humidity) for 7d. Thereafter, two specimens were stacked using the CABR-NES contact shrinkage deformation tester (Figure 4) and bonded with gaskets above the specimen. The dial head was in contact with the gasket to detect changes in specimen length. The samples were tested under normal curing conditions (RH = 92%, T = 20 °C), and the readings of the dial indicator and the sample mass were recorded for 40 days. The average of the linear shrinkage was used to represent the change in sample height, and the linear shrinkage formula is shown in Equation (1). The change in sample mass was used to characterize the water-loss rate of the material, and the water-loss rate formula is shown in Equation (2).

(1) $$e_{sl}=\frac{R_{t}-R_{0}}{H_{0}}\times 100$$

$e_{sl}$—Linear shrinkage, accurate to 0.01%;
$H_{0}$—Original height of sample (mm);
$R_{0}$—Initial readings of dial indicator (mm);
$R_{t}$—Dial indicator readings of contraction process at some time (mm).

(2) $$\omega =\left(1-\frac{m_{t}}{m_{s}}\right)\times 100$$

ω—Water loss rate, accurate to 0.01%;
$m_{t}$—Sample quality of at during shrinkage some time (g);
$m_{s}$—The original quality of sample (g).

#### 2.2.4. XRD and SEM

The mineral composition test was to take a representative sample block from the test sample and first soak it in anhydrous ethanol for 1 h to stop the hydration process. Then the sample block was dried at T = 45 °C. Finally, the sample powder was ground with a mortar pestle and filtered through a 200-mesh sieve. Then it was scanned for 20 min with an X-ray diffractometer (PANalytical. B.V, Almelo, The Netherlands) to identify hydration products in cement-CFBS stabilized clay. The microstructure and hydration phase of the cured samples were identified by scanning electron microscopy (Zeiss, Jena, Germany).

## 3. Results

### 3.1. Mechanical Properties

**Figure 5:**
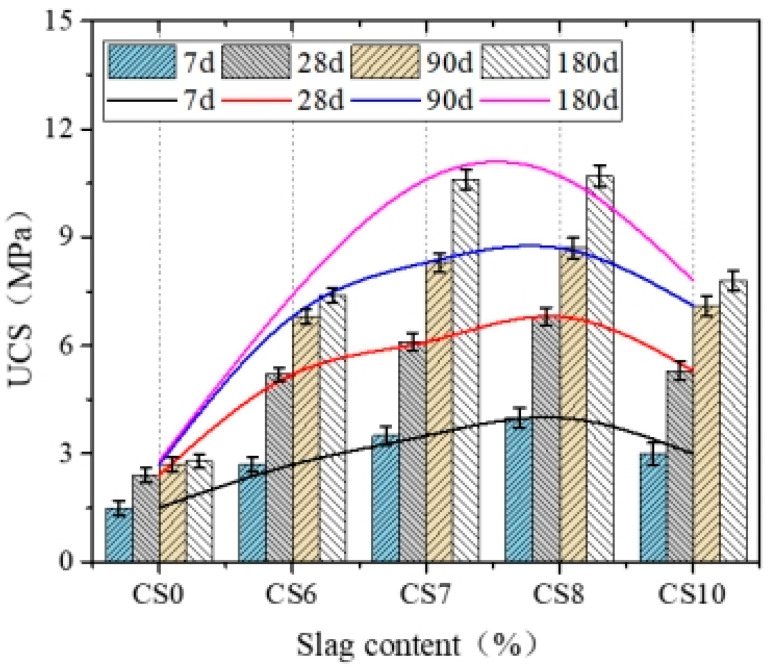
Effect of CFBS content on the UCS of cement-stabilized clay. The error bars represent the standard deviation of the test results for five samples.

**Figure 6:**
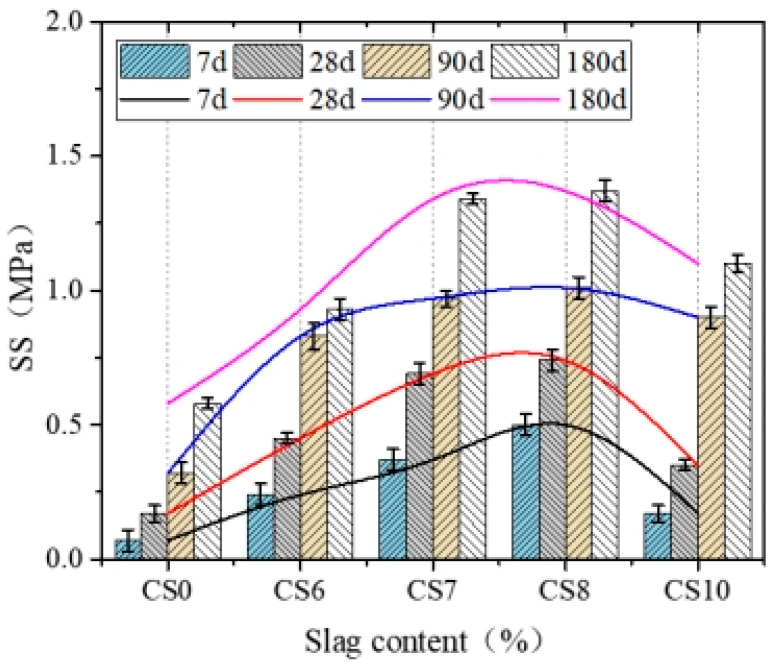
Effect of CFBS content on the SS of cement-stabilized clay. The error bars represent the standard deviation of the test results for five samples.

Figure 5 and Figure 6 illustrate the UCS and SS of cement–CFBS-stabilized clay.

Figure 5 and Figure 6 show the strength results of cement-CFS-stabilized clay. Figure 5 shows that CFBS can significantly improve the UCS of cement-stabilized clay. With the increase of CFBS, the UCS of cement–CFBS-stabilized clay material first increased and later decreased and was higher than that of cement-stabilized clay (CS0); it reached a maximum value when CFBS addition was 80% (CS8). Compared with CS0, the 7d, 28d, 90d, and 180d UCS of CS8 was found to have increased by 166%, 258%, 222%, and 257%, respectively. The 90d UCS of CS0 was stable however, the UCS of cement–CFBS-stabilized clay continued to grow after 90d, and the later strength was greater. A faster strength gain was also observed by Ana Paula Furlan et al. [2].

As the cement content was only 6%, the specific surface area of soil particles was much larger than that of cement, and the gel products formed by the cement could not fully connect the soil particles thus, the UCS was low [18,19,20]. CFBS is granular and has a continuous gradation. In an early stage, it mainly provides a skeleton for clay, and when the content of soil particles is 70–80%, the pores of the CFBS are filled, and the early strength is improved significantly. CFBS contains f-CaO and anhydrite, which provides more Ca^2+^ and produces more network ettringite. Dongxing Wang et al. [21] also found that the strength was improved due to the physical–chemistry effects of slag particles, such as local cementation. In addition, CFBS content was high, fully dispersed in soil, formed a large number of crystal nucleus points, and the fine slag particle sulfur calcium content was high during the 7d–28d rapid reaction, so the 7d–28d strength increases greatly, and, later, large particle slag gradually participates in the reaction, so the strength continues to grow [22].

Figure 6 showed that the change rule of SS with CFBS addition is the same as that of UCS, and SS reaches the peak when the CFBS addition is 80% (CS8), and then decreases, the difference was that the improvement was more obvious than with CS0. Compared with CS0, the 7d, 28d, 90d, and 180d, SS of CS8 was found to have increased by 614%, 335%, 216%, and 136%, respectively. Ivana Barišić et al. [23] observed that the amount of slag used often significantly affects the SS of slag-containing mixtures, similar to the compressive strength, as mentioned earlier. This is because UCS is jointly determined by the inter-skeleton effect, the hydration products of cement and CFBS, and the bonding between the mixtures. The main reason for SS growth is the cementation product [19]. With an increase in CFBS content and age, f-CaO and anhydrite react with volcanic ash to form CSH and CAH gel substances. These spread between soil particles, increasing the internal bond strength of cement-stabilized clay and the strength of the transition zone at the interface between cement and slag.

### 3.2. Resistance to Water Immersion

**Figure 7:**
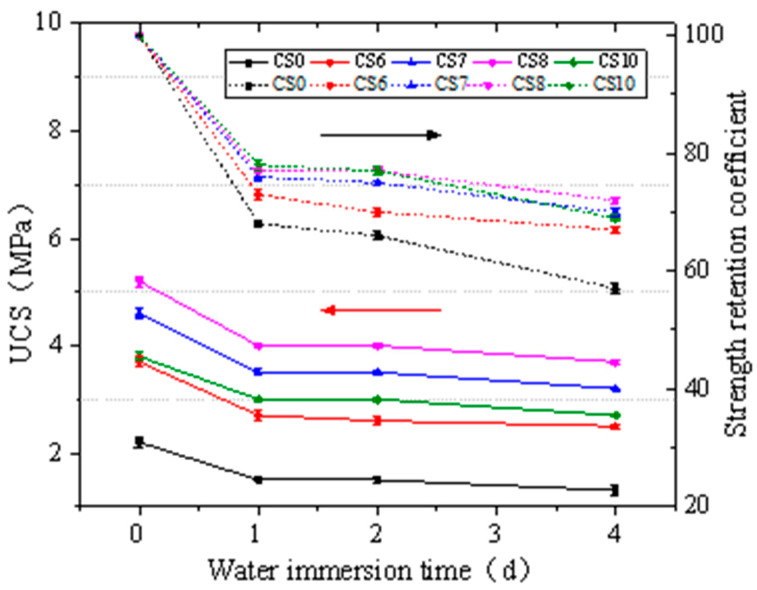
Influence of water immersion on UCS of stabilized clay of cement CFBS. The error bars represent the standard deviation of the test results for five samples.

The effect of CFBS on the UCS of cement-stabilized clay after water immersion is presented in Figure 7.

Figure 7 showed that the rules of water immersion resistance of cement-stabilized clay after adding CFBS. The UCS of cement-stabilized clay (CS0) decreased from 2.2 MPa to 1.3 MPa with the increase of immersion days. Similarly, the strength retention coefficient decreased from 100% to 57%. For cement–CFBS-stabilized clay, taking CS8 as an example, UCS decreased from 5.7 MPa to 3.7 MPa, and the strength retention coefficient decreased from 100% to 72%. This shows that CFBS can significantly improve the water stability of cement-stabilized clay. Dongxing Wang et al. proposed that the change of water stability of cement-CFS-stabilized clay can be explained from three aspects: The repulsion between clay particles increases, the chemical bonding interface is damaged, and the formation of hydration products. Taking CS8 as an example, the decreasing rate of UCS after 1–2d immersion is related to the formation of hydration products. CSH gel is produced by hydration of cement and CFBS. The generated hydration products can fill the pores and prevent water intrusion, so as to protect the chemical interface from damage, and promote the finite enhancement and densification of the skeleton structure of cement–CFBS-stabilized clay during immersion. However, after 4d immersion, since most of the water was absorbed by the specimen and filled into the pore spaces, the repulsive force between clay particles was enhanced, the pores became larger, and the strength greatly decreased.

### 3.3. Shrinkage Properties

**Figure 8:**
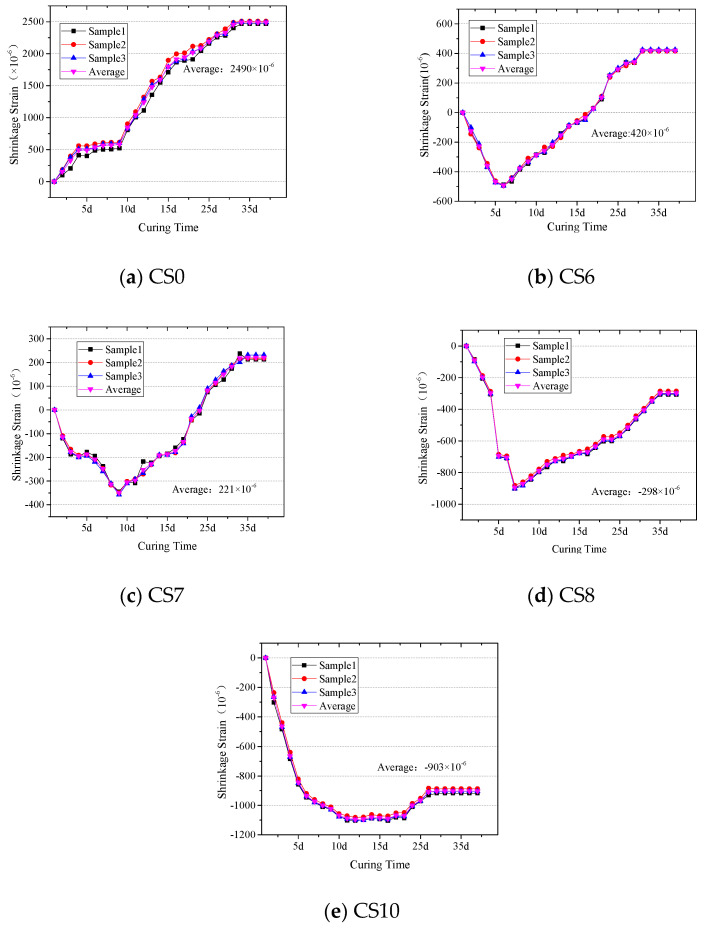
The shrinkage strain of CS0, CS6, CS7, CS8, CS10 (**a**–**e**, respectively)

Figure 8 and Table 4 show that the average shrinkage strain gradually changes with the increase of CFBS content. From the results, the shrinkage strain of cement-stabilized clay (CS0) increases with curing time; Apinum Buritatum et al. also observed macro and micro cracks [24]. After adding 60–80% CFBS, Figue 8b–d showed that CS6, CS7, and CS8 showed a slight increase in the first 10d. After that, CS6 and CS7 began to shrink, and the shrinkage strain was zero at 35d. The strain change trend of CS8 was the same, but it showed a slight expansion when it was stable, and the strain was 297.5 × 10^−6^. As an extreme, if CFBS completely replaces clay, CS10 shows continuous expansion with the extension of curing time and reaches expansion peak at 10d, and later dehydration shrinkage has little effect on the strain. It can be seen that the expansion material introduced by CFBS is the fundamental reason for the inhibition of cement-stabilized clay shrinkage. The expansive substances may be Ca (OH)_2_, formed by f-CaO digestion in CFBS; dihydrate gypsum, formed by anhydrite hydrolysis; and ettringite, formed by gypsum reacting with active aluminum in an alkaline environment [25].

**Figure 9:**
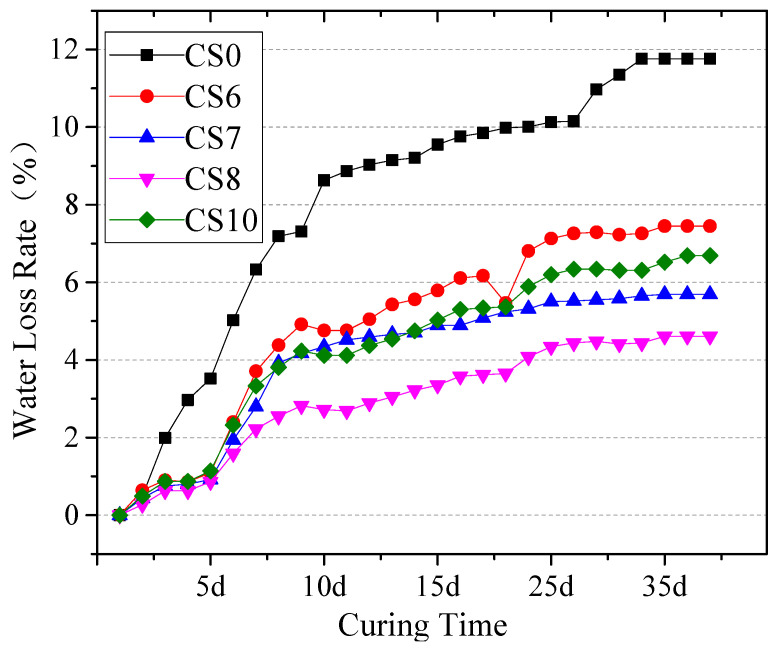
The water loss rate of CS0, CS6, CS7, CS8, CS10.

Figure 9 shows that the OMC of cement–CFBS-stabilized clay is higher than that of cement-stabilized clay (Table 3). However, the water loss rate of CS6–CS10 was lower than that of CS0. The water absorbed by CFBS was not completely released into the air. The water was digested by f-CaO, hydrolyzed by anhydrite, and utilized by cement hydration to form synthetic water. However, the content of CFBS in CS10 was the highest, which contained the most f-CaO and anhydrite. The water loss rate was higher than CS8 and it can be seen that the water requirement of the raw materials is only one of the factors affecting the water loss rate. This is also related to the structure of the mixture Lin Min et al. observed in which the water loss rate is related to the mixture structure [26]. Combined with the relationship between the water loss rate and strength, it can be speculated that, when the slag content is 70–80%, the mixture is densest and the porosity is smallest, so the strength is high and the water-loss rate is low, which is an indirect cause of CFBS inhibiting shrinkage.

### 3.4. Microstructure

**Figure 10:**
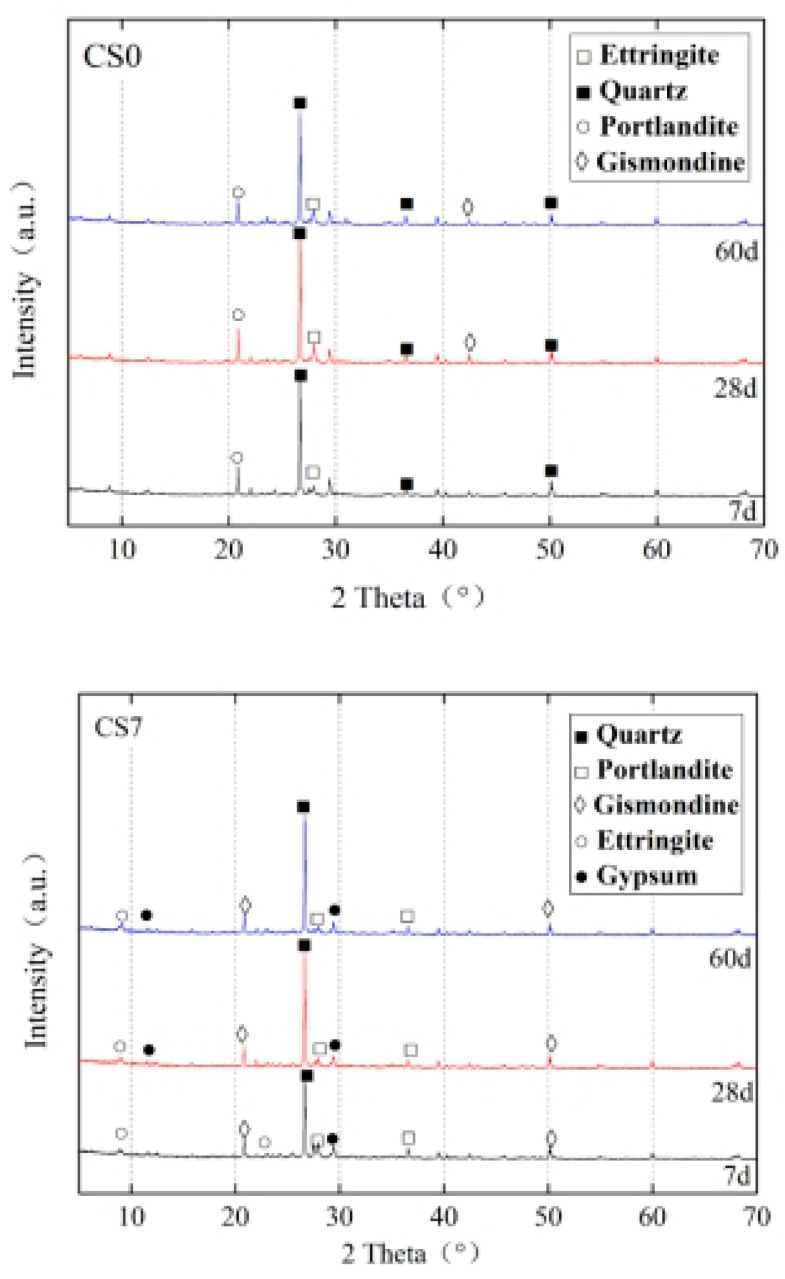
The XRD patterns of cement-stabilized clay (CS0) and cement–CFBS-stabilized clay (CS7).

**Figure 11:**
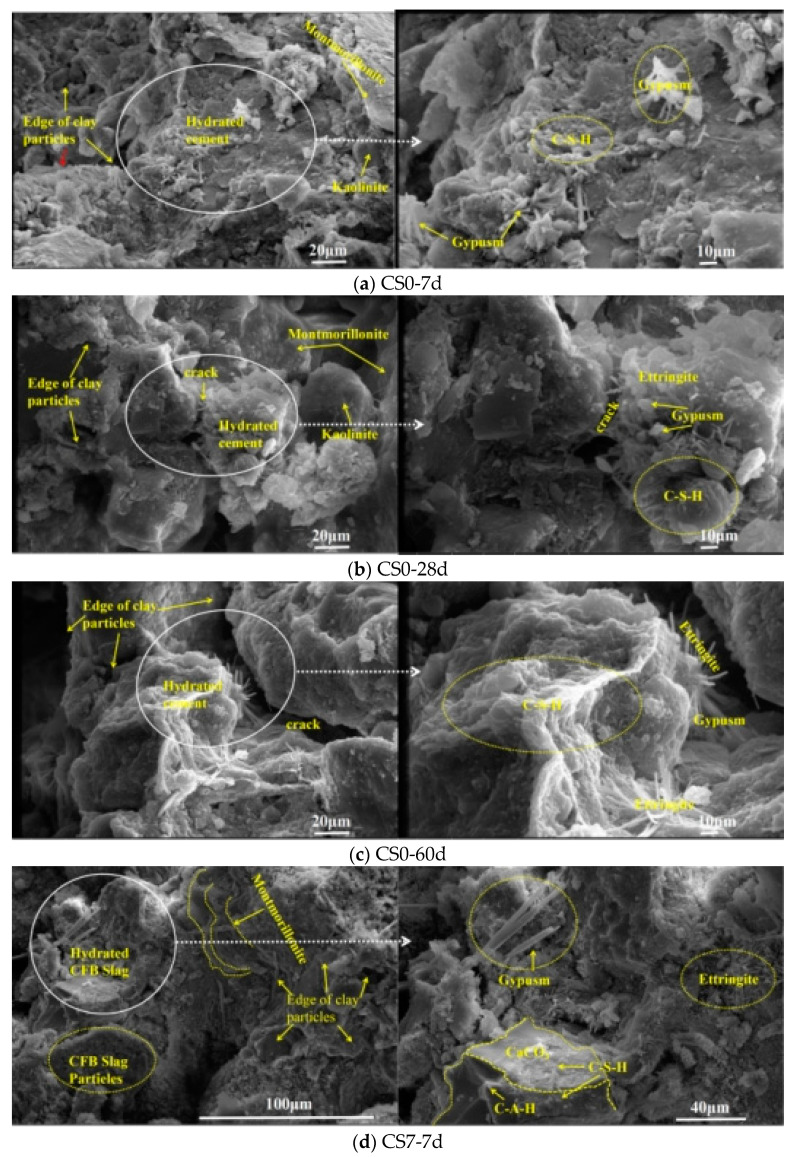

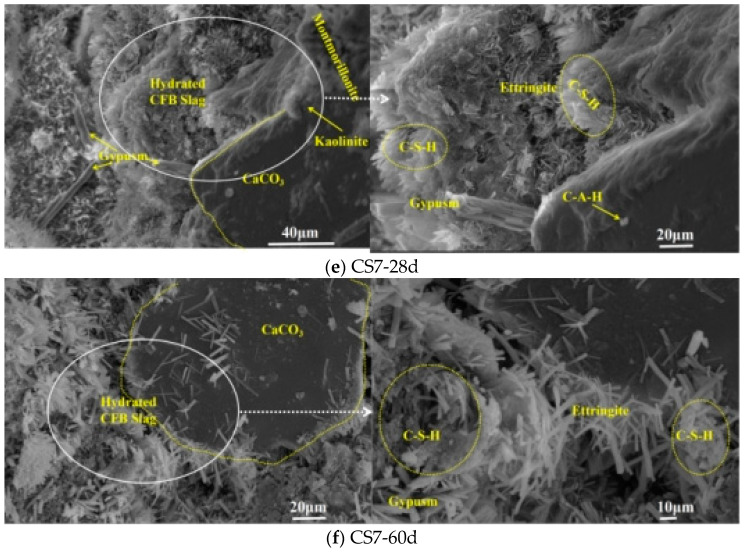
The SEM images of cement-stabilized clay (CS0, cement–CFBS-stabilized clay (CS7) at 7d, 28d, and 60d. (**a**) The SEM image of CS0-7d; (**b**) The SEM image of CS0-28d; (**c**) The SEM image of CS0-60d; (**d**) The SEM images of CS7-7d; (**e**) The SEM images of CS7-28d; (**f**) The SEM images of CS7-60d.

**Figure 12:**
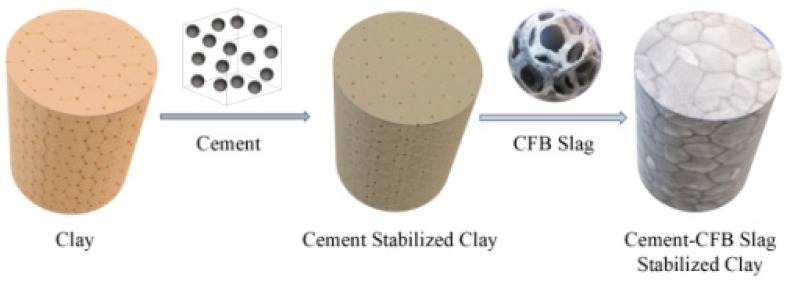
Crystal model of cement(–CFB)-slag-stabilized clay.

Figure 10, Figure 11 and Figure 12 show the XRD, SEM, and model diagrams of CS0 and CS7. Figure 10 shows that the mineral component of CS0 were mainly Ca (OH)_2_ and ettringite. The main mineral component of CS7 also includes gypsum. With the increase of time, the consumption of Ca (OH)_2_ and increase of ettringite are accelerated. This shows that the cement content in CS0 was too small. Moreover, CFBS has higher volcanic ash activity and can consume more Ca (OH)_2_. It can be seen from Figure 1 that CFBS contains more active Al_2_O_3_, CaO, and anhydrite, so more Aft can be generated in cement–CFBS-stabilized clay. Mingkai Zhou et al. [16] also found that CSH gel was difficult to observe when studying CFBCA, but it can be seen that there are many dispersion peaks of CS7 and the amount of CSH gel increases.

Figure 11 showed that with the increase of time, the structures of CS0 and CS7 are gradually denser. Compared with CS0, the structure of CS7 is more compact. There was a small amount of CSH gel on the surface of soil particles in CS0 at 7d (Figure 11a). There are more acicular columnar hydration products and CSH gel at 28d and 60d (Figure 11b,c), but there are still more pores. At 7d, there are needle-like columnar products and cubic crystals in CS7 (Figure 11d), which are ettringite, gypsum, and CaCO_3_. These are mainly from the hydration of anhydrite, CaO, SiO_2_, and Al_2_O_3_ in CFBS. At 28d, the surface of the CFBS particles in CS7 was wrapped by a large number of gel products from CSH (Figure 11e). The surface of the CFBS particles in CS7 was surrounded by a large number of needle-like products and CSH gel products at 60d (Figure 11f), which indicates that the surfaces of the CFBS particles were gradually corroded from hydration, and generated more hydration products, mainly from the volcanic ash reaction of CFBS. At the same time, it can be seen that CFBS can produce more CSH gel and Aft in cement-stabilized clay, and the internal structure tends to be dense. This shows that the hydration products generated by CFBS fill the internal pores, promoting the development of structural densification. Dongxing Wang et al. [24] also found that slag particles had the effect of filling pores. Mingkai Zhou et al. [16] found that the hydration products of CFBCA were easy to dissolve and promote structural densification.

Therefore, the hydration products of CFBS (CSH gel, AFt) play an important role in structural densification, strength development, and shrinkage inhibition. This is mainly due to, firstly, CFBS promoting structural densification, as water evaporation is not easy and water loss rate decreases, thereby inhibiting shrinkage. Secondly, Figure 1 shows that CFBS contains CaO and anhydrite. Ca (OH)_2_ generated by CaO hydration provides an alkaline environment for the dissolution and hydration of CFBS [16,26]. After that, ettringite is generated, and the volume becomes larger. Meanwhile, anhydrite absorbs water to generate gypsum, and the crystal volume becomes larger, resulting in macroscopic expansion of the specimen. Therefore, the shrinkage deformation caused by water loss is compensated therefore CFBS improves the strength and shrinkage of cement-stabilized clay.

## 4. Conclusions

The effects of different dosages of CFBS on the strength, water stability, and shrinkage performance of cement stabilized clay were studied. The reaction mechanism of CFBS improving cement stabilized clay was revealed by XRD and SEM. The conclusions are as follows:

(1) The strength (UCS, SS) of cement-CFBS-stabilized clay increases first and then decreases with the increase of CFBS content. When CFBS content is 80% (CS8), it reaches the peak. The unconfined compressive strength monotonically increases with age. UCS reached 10.7 MPa at 180d;
(2) CFBS as a cement-stabilized clay admixture can significantly improve water stability. The water immersion resistance of cement–CFBS-stabilized clay increases first and then decreases with an increase in CFBS content. When the CFBS content is 70–80%, water immersion resistance was best. For shrinkage performance, the volume stability of cement–CFBS-stabilized clay was the best when CFB slag content was 70%;
(3) The reaction mechanism of CFBS in cement-stabilized clay has two aspects: (a) The active silicon and aluminum substances in CFBS are easily dissolved, which accelerates the formation of CSH and AFt, and makes the structure dense and causes the strength increase. (b) The volumes of AFt and gypsum increase in the generation process, causing the mixture to expand, making up for the volume shrinkage and improving the strength and stability of cement-stabilized clay;
(4) At present, current research focuses on cement-stabilized clay prone to water loss and shrinkage cracking, and CFBS has expansibility, it cannot be widely used. However, we studied the inhibition mechanism of CFBS on the shrinkage of cement-stabilized clay and the improvement mechanism of mechanical properties, providing theoretical and reference for the use of CFBS. However, the cement and CFBS systems are complex and the base materials include gravel, etc. Future research needs to study the inhibitory effect of CFBS on the shrinkage of cement-stabilized base materials. In addition, the test standard of cement-CFBS stabilized clay should be established to make CFBS resource utilization.

## Figures and Tables

**Table 1:** Chemical composition of CFBS/%.

Sample	SiO_2_	Fe_2_O_3_	CaO	MgO	SO_3_	Loss	f-CaO
CFBS (%)	46.6	3.6	7.1	0.6	6.4	3.7	3.63

**Table 2:** Properties of cement used.

Cement Type	Specific Surface Area/m^2^·kg^−1^	Standard Consistency/%	Setting Time/Min		Flexural Strength/MPa		Compressive Strength/MPa	
			Initial Setting	Final Coagulation	7d	28d	7d	28d
**Zhuoyue PSA32.5**	223	30.6	201	266	5.5	8.0	25.5	43.3

**Table 3:** Mix proportion and compaction test results.

Sample	Mass Fraction of Different Materials/%			Compaction Test Results	
	Cement	Clay	CFBS	Maximum Dry Density/g·cm^−3^	Optimum Moisture Content/%
CS0	6	100	0	1.895	12.7
CS6	6	40	60	1.760	13.2
CS7	6	30	70	1.754	14.5
CS8	6	20	80	1.740	15.9
CS10	6	0	100	1.600	18.6

**Table 4:** The shrinkage strain, average value of shrinkage strain, and coefficient of variation for the samples on the 40th day of the test.

Addition of CFBS (%)	Sample No.	Shrinkage Strain (10^−6^)	Average (10^−6^)	Dispersion Coefficient (CV)
0%	1	2470	2490	0.7
	2	2509		
	3	2491		
60%	1	419	420	1.1
	2	416		
70%	3	425		
	1	213	221	4.5
	2	218		
	3	232		
80%	1	−307	−298	3.9
	2	−285		
	3	−302		
100%	1	−917	−903	1.6
	2	−887		
	3	−905		

## Data Availability

The data used to support the findings of this study can be made available from the corresponding author upon request.

## References

[1] Mola-Abasi H., Shooshpasha I. (2016). Influence of zeolite and cement additions on mechanical behavior of sandy soil. J. Rock Mech. Geotech. Eng..

[2] Paula F.A., Andry R., Harifidy R., Ouali A., Daniel L., Dimitri D. (2020). Effect of fly ash on microstructural and resistance characteristics of dredged sediment stabilized with lime and cement. Constr. Build. Mater..

[3] Liu H., Zhao J., Wang Y., Yi N., Cui C. (2021). Strength Performance and Microstructure of Calcium Sulfoaluminate Cement-Stabilized Soft Soil. Sustainability.

[4] Wang J., Li X., Wen H., Muhunthan B. (2020). Shrinkage cracking model for cementitiously stabilized layers for use in the mechanistic-empirical pavement design guide. Transp. Geotech..

[5] Kangni-Foli E., Poyet S., Le Bescop P., Charpentier T., Bernachy-Barbé F., Dauzères A., L’Hôpital E., d’Espinose de Lacaillerie J.-B. (2021). Carbonation of model cement pastes: The mineralogical origin of microstructural changes and shrinkage. Cem. Concr. Res..

[6] Wang J., Wen H., Muhunthan B. (2020). Development of test methods to characterize the shrinkage properties of cementitiously stabilized materials. Transp. Geotech..

[7] Xue Y., Hou H., Zhu S., Zha J. (2008). Utilization of municipal solid waste incineration ash in stone mastic asphalt mixture: Pavement performance and environmental impact. Constr. Build. Mater..

[8] Zhang S., Yang X., Xie S., Yin P. (2020). Experimental study on improving the engineering properties of coarse grain sulphate saline soils with inorganic materials. Cold Reg. Sci. Technol..

[9] Subramanian S., Khan Q., Ku T. (2020). Effect of sand on the stiffness characteristics of cement-stabilized clay. Constr. Build. Mater..

[10] Xu F., Wei H., Qian W., Chen X., Xu T., He Y., Wen G. (2020). Experimental investigation on replacing cement by sintered limestone ash from the steelmaking industry for cement-stabilized soil: Engineering performances and micro-scale analysis. Constr. Build. Mater..

[11] Amini O., Ghasemi M. (2019). Laboratory study of the effects of using magnesium slag on the geotechnical properties of cement stabilized soil. Constr. Build. Mater..

[12] Goodarzi A.R., Salimi M. (2015). Stabilization treatment of a dispersive clayey soil using granulated blast furnace slag and basic oxygen furnace slag. Appl. Clay Sci..

[13] Xiaoyuan W., Pengju H., Xiaohong B., Xiangyu L. (2019). Influences of slag on properties of lightweight cement-treated soils subjected to sulfate corrosion. Constr. Build. Mater..

[14] Lin M., Lu X., Wang Q., Pan Z., Hong Y., Ji X. (2014). The experimental study of fly ash recirculation combustion characteristics on a circulating fluidized bed combustor. Fuel Process. Technol..

[15] Anthony E.J., Berry E.E., Blondin J., Bulewicz E.M., Burwell S. (2003). Advanced ash management technologies for CFBC ash. Waste Manag..

[16] Zhou M., Chen P., Chen X., Ge X., Wang Y. (2020). Study on hydration characteristics of circulating fluidized bed combustion fly ash (CFBCA). Constr. Build. Mater..

[17] Lee H.K., Jeon S.-M., Lee B.Y., Kim H.-K. (2020). Use of circulating fluidized bed combustion bottom ash as a secondary activator in high-volume slag cement. Constr. Build. Mater..

[18] Jun W., Li L., Yongfeng D., Guoping Z., Annan Z., Qiong W. (2020). Distinguishing the effects of cementation versus density on the mechanical behavior of cement-based stabilized clays. Constr. Build. Mater..

[19] Zhang J., Weng X., Liu J., Liu W., Gao R., Lin K. (2014). Experimental research on mechanical property and water stability of complex stabilized sandy soil. Mater. Rep..

[20] Wei Y., Faqin D., Yuequan D., Ping H. (2013). Influence of different pretreatment methods on hydrothermal synthesis of calcium sulfate whisker from CFBC ash and slag. Mater. Rep..

[21] Wang D., Gao X., Liu X., Zeng G. (2021). Strength, durability and microstructure of granulated blast furnace slag-modified magnesium oxychloride cement solidified waste sludge. J. Clean. Prod..

[22] Zhou M., Cheng X., Chen X. (2021). Studies on the Volumetric Stability and Mechanical Properties of Cement-Fly-Ash-Stabilized Steel Slag. Materials.

[23] Barišić I., Dimter S., Rukavina T. (2014). Strength properties of steel slag stabilized mixes. Compos. Part B.

[24] Buritatum A., Horpibulsuk S., Udomchai A., Suddeepong A., Takaikaew T., Vichitcholchai N., Horpibulsuk J., Arulrajah A. (2021). Durability improvement of cement stabilized pavement base using natural rubber latex. Transp. Geotech..

[25] Li X., Chen Q., Huang K., Ma B., Wu B. (2012). Cementitious properties and hydration mechanism of circulating fluidized bed combustion (CFBC) desulfurization ashes. Constr. Build. Mater..

[26] Lin M., Wang W., Li Y. (2018). Preparation of inorganic compound early strength agent and its effect on properties of cement stabilized macadm material. Mater. Rep..

